# Is absorption of irrigation fluid a problem in Thulium laser vaporization of the prostate? A prospective investigation using the expired breath ethanol test

**DOI:** 10.1186/s12894-015-0029-2

**Published:** 2015-04-24

**Authors:** Livio Mordasini, Dominik Abt, Gautier Müllhaupt, Daniel S Engeler, Andreas Lüthi, Hans-Peter Schmid, Christoph Schwab

**Affiliations:** Department of Urology, Cantonal Hospital St. Gallen, Rorschacherstrasse 95, 9007 St. Gallen, Switzerland; Department of Anaesthesiology, Cantonal Hospital St. Gallen, Rorschacherstrasse 95, 9007 St. Gallen, Switzerland

**Keywords:** Prostate, Benign prostatic hyperplasia, Transurethral laser vaporization of the prostate, Transurethral resection syndrome, Absorption irrigation fluid, Expired breath ethanol test

## Abstract

**Background:**

Benign prostatic hyperplasia (BPH) is a prevalent entity in elderly men. If medical treatment fails, monopolar transurethral resection of the prostate (TUR-P) is still considered as the standard treatment. The proportion of high-risk patients with cardiac comorbidities increases and TUR-P goes along with a relevant perioperative risk. Especially large volume influx of irrigation fluid and transurethral resection syndrome (TUR syndrome) represent serious threats to these patients. Using isotonic saline as irrigation fluid like in transurethral laser vaporization (TUV-P), TUR syndrome can be prevented. However, no prospective trial has ever assessed occurrence or extent of irrigation fluid absorption in Thulium Laser TUV-P.

**Methods/Design:**

This is a single-center prospective trial, investigating, if absorption of irrigation fluid occurs during Thulium Laser TUV-P by expired breath ethanol test. The expired breath ethanol technique is an established method of investigating intraoperative absorption of irrigation fluid: A tracer amount of ethanol is added to the irrigation fluid and the absorption of irrigation fluid can be calculated by measuring the expiratory ethanol concentrations of the patient with an alcohol breathalyzer.

Fifty consecutive patients undergoing TUV-P at our tertiary referral center are included into the trial. Absorption volume of irrigation fluid during Thulium Laser TUV-P is defined as primary endpoint. Pre- to postoperative changes in bladder diaries, biochemical and hematological laboratory findings, duration of operation and standardized questionnaires are assessed as secondary outcome measures.

**Discussion:**

The aim of this study is to assess the safety of Thulium Laser TUV-P in regard to absorption of irrigation fluid.

## Background

Benign prostatic hyperplasia (BPH) is a prevalent entity, affecting over 50% of men older than 60 years [1]. The clinical picture of the disease includes lower urinary tract symptoms such as interrupted and weak urinary stream, nocturia, urgency, leaking and even sexual dysfunction in some individuals [2]. Medical therapy is usually the first-line treatment [3]. However, the efficacy of drugs like alpha-blockers is limited, and as disease progresses, more invasive treatment options have to be taken into consideration.

In cases with moderate to severe lower urinary tract symptoms (LUTS) monopolar transurethral resection of the prostate (TUR-P) is still the standard treatment. Especially in frail patients, conventional TUR-P is associated with relevant and potentially deleterious complications [4-6].

The proportion of elderly patients on anticoagulation or antiplatelet therapy with cardiac comorbidities increases. Especially major bleeding and transurethral resection syndrome (TUR syndrome) put these high-risk patients at a relevant perioperative risk.

TUR syndrome is caused by absorption of electrolyte-free irrigating fluid (which has to be used in monopolar TUR-P), and consists of symptoms of the circulatory and nervous systems. Mild forms are common and often go undiagnosed, while severe forms of the TUR syndrome are potentially life-threatening [7].

Using isotonic saline, like in bipolar TUR-P and TUV-P, TUR syndrome can be prevented. Moreover, these techniques were thought to completely prevent influx of irrigation fluid into the vascular system due to their excellent coagulation properties [8-11].

However, significant intraoperative fluid absorption has been demonstrated in bipolar TUR-P and with the Greenlight-Laser. The authors concluded, that these techniques should be used with caution in patients with significant cardiovascular comorbidities [12,13].

Ethanol monitoring was first used in the late 1980’s as an alternative to traditional methods of measuring fluid absorption (i.e. measuring volumetric fluid balance and serum sodium concentration). These techniques, however, are bothersome and must be carried out meticulously to yield a valid Figure of absorption [14]. If ethanol is added to the irrigation fluid as a tracer, the volume of fluid absorbed can be estimated from the amount of ethanol measured in the patient’s exspired breath.

The expired breath ethanol technique is an established method of investigating intraoperative absorption of irrigation fluid [15]. Isotonic 0.9% saline including 1% of ethanol is used for intra-operative irrigation. The absorption of irrigation fluid can be estimated by measuring the expiratory ethanol concentrations with an alcohol breathalyzer.

During the last years, the Thulium laser has emerged as an alternative to other types of lasers, combining the best features for performing vaporization techniques: Thulium laser has a wavelength of 2013 nm, and its target chromophore is water. The energy of the Thulium laser has a high tissue absorption rate, producing effective vaporization with scant depth in the remaining tissue. As the properties of water remain unaltered until it reaches boiling point, the effect of the laser on the tissue remains constant throughout the surgical procedure [11].

The short-term complication rate with the Thulium laser is similar to the rate described after vaporization with other laser systems [16-21] and less than that with TUR-P. Thus, Thulium vaporization of the prostate has established as a standard procedure in many urological departments including ours.

Despite recent publications on the safety and complications of Thulium vaporization of the prostate, to our knowledge, no prospective trial has assessed occurence and extent of irrigation fluid absorption in TUV-P using Thulium Laser.

We therefore aim to investigate the relevance of irrigation fluid absorption during Thulium Laser vaporization of the prostate by expired breath ethanol test.

## Methods and design

### Study design and location

This is a single-center prospective trial conducted at the urological department of Cantonal Hospital St. Gallen, St. Gallen, Switzerland.

### Study population and recruitment

Recruitment of the study participants is performed at the urological outpatient clinic of Cantonal Hospital St. Gallen by the principle investigator (PI). The PI will check for inclusion and exclusion criteria (Table 1) by reviewing the patient’s medical record and by patient-doctor conversation. Study participants are thoroughly informed about the study and included into the trial if informed consent is given.

**Table 1:** **Inclusion and exclusion criteria**

**Inclusion criteria**	**Exclusion criteria**
• Men older than 40	• Mild symptoms (IPSS <8)
• Patient must be a candidate for TUV-P	• Urethral stenosis
• Refractory to medical therapy or patient is not willing to consider further medical treatment	• Bladder diverticulum (>100 ml)
	• Former alcoholic or chronic liver
• Written informed consent	• disease

### Study procedures

**Figure 1:**
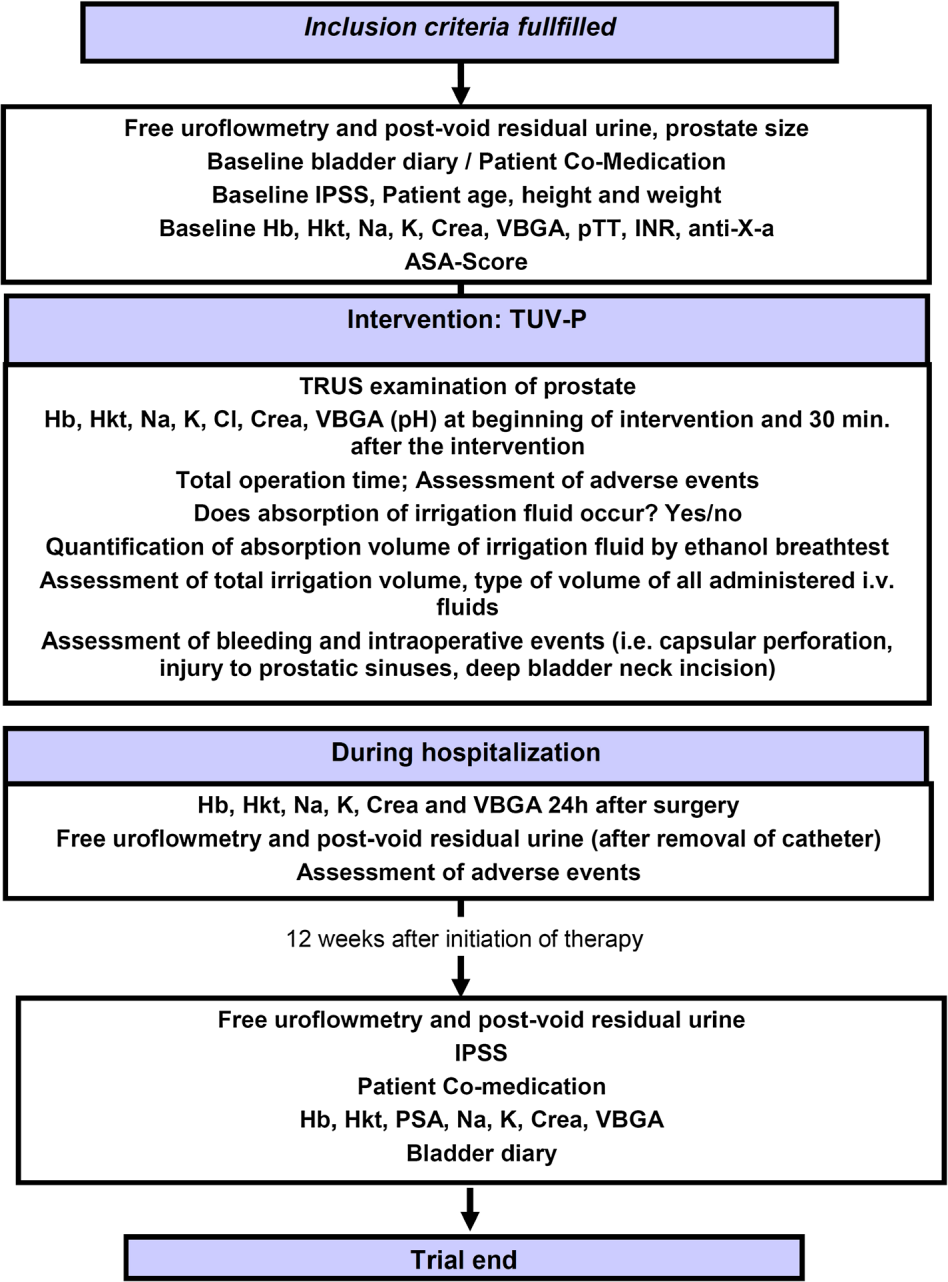
Timetable and characteristics of study visits.

Age, height, weight, free uroflowmetry, post void residual urine, co-medication, bladder voiding diary, International Prostate Symptom Score (IPSS) and ASA Score are determined preinterventionally in all study participants (Figure 1). The intervention is performed in an inpatient setting.

### TUV-P

Before the operation starts, prostate size is assessed by transrectal ultrasound measurement (TRUS).

In the trial setting, TUV-P is only performed by surgeons with an experience of more than 100 transurethral interventions.

A Thulium continuous wave laser is employed (Revolix Duo 120 W, 2 micron thulium continuous wave laser; LISA laser products OHG, Katlenburg, Germany).

Application is carried out with a 24-Chr. continuous flow cystoscope (Karl Storz Endoskope; Anklin AG, Binnigen, Switzerland) and a 550-μm front-firing laser fiber. The procedure begins at 80-W. In the first phase of the surgery, the bladder neck is opened and the median lobe vaporized. The lateral lobes are then vaporized until a working channel is created in the prostate. A power of 120-W is used from then on, completing vaporization of the median and lateral lobes. At the end of surgery, a three-way 20-Chr. silicone urethral catheter will be inserted and continuous bladder irrigation with saline solution is maintained. Surgery will be performed under spinal or general anesthesia, according to anesthetist’s and patient’s preferences.

Bleeding or other intraoperative events such as capsular perforation, injury to prostatic sinuses or deep bladder neck incision are documented by the surgeon.

### Expired breath ethanol test

Bladder irrigation is performed with conventional isotonic 0.9% saline with 1% ethanol added (Somanol® irrigation fluid Ecobag 5000 ml, Braun Medical AG, Sempach, Switzerland). The end-expiratory ethanol concentrations are measured at the beginning of the operation and at regular 10-min intervals (5-min intervals if ethanol is detected) throughout the procedure using an electrochemical AlcoQuant 6020 alcohol breathalyzer (Envitec GmbH, Wismar, Germany).

The values obtained represent the corresponding blood ethanol concentrations in mg/mL. A blood ethanol concentration of 0.05 mg/mL corresponds to ~150 mL fluid absorption. If patients are under general anesthesia, the alcohol breathalyzer is intermittently connected to the endotracheal tube via an adapter (this adapter is included in the AlcoQuant 6020 breathalyzer kit) after insufflation of the lungs with a resuscitation bag. The passively expired air is then used for the measurements. Patients under spinal anesthesia are asked to expire directly into the alcohol breathalyzer.

Hahn’s mathematical formula (pre-programmed on a pocket calculator) will be used for the quantification of the total absorption volume intraoperatively [22].

$$ Ab{s}_{tot}={\displaystyle \sum }7007\  EB- ethano{l}_i \times \varDelta EB- ethano l + 632\  EB- ethano{l}_i+202 $$

### Blood analysis

After their baseline assessment on the day before the operation, hemoglobin, hematocrit, sodium, potassium, creatinine and a venous blood gas analysis are repeated at the beginning of the intervention, 30 minutes and 24 hours after the intervention to document possible electrolyte shifts.

## Study outcome measures

### Characteristics and timing of visits

After hospital discharge, a regular follow-up control is planned after 12 weeks assessing free uroflowmetry, post void residual, bladder voiding diary and IPSS. In addition, hemoglobin, hematocrit, sodium, potassium, creatinine and a venous blood gas analysis are determined.

### Primary and secondary endpoints

Absorption volume of irrigation fluid during the Thulium laser TUV-P intervention was defined as primary endpoint. Secondary study endpoints are shown in Table 2.

**Table 2:** **Primary and secondary endpoints**

Primary endpoint	• Absorption volume of irrigation fluid during TUV-P
Secondary endpoints	• Duration of surgery
	• Assessment of bleeding and relevant intraoperative events (capsular perforation, injury to prostatic sinuses or deep bladder neck incision) by the surgeon
	• Amount of laser energy used intraoperatively (kilojoules)
	• Pre- to postoperative (30 min after intervention) changes in serum biochemical and hematological variables (creatinine, sodium, potassium, chloride), venous pH, hemoglobin
	• Pre- to postoperative changes (12 weeks after intervention) in flow and residual urine
	• Total irrigation volume and volume of all administered i.v. fluids
	• Duration of hospitalization post procedure
	• Duration of post procedure catheterization
	• Pre- to postoperative changes (12 weeks after intervention) in the IPSS
	• Pre- to postoperative changes (12 weeks after intervention) in bladder diary

## Statistics, study sample size and power calculation

### Determination of sample size

According to Wettstein et al., approximately 44% of patients showing a positive ethanol test were supposed. Among those, median fluid absorption was 1249 ml (138 – 2452 ml) [13]. This would indicate a mean fluid absorption of approximately 1250 ml, with standard deviation 579 ml. To estimate a 95% confidence interval for fluid absorption with width no larger than +/− 250 ml, we would therefore require 23 patients showing a positive ethanol test, indicating approximately evaluable 50 patients in total must be recruited.

For the secondary endpoint of rate of positive ethanol tests, the 95% confidence interval should therefore be no wider than +/− 14%.

### Planned analyses

All study participants undergoing TUV-P will be considered in the final data analysis.

### Primary analysis

Fluid absorption will be summarized as mean (95% confidence interval), including only those patients with a positive ethanol test.

### Secondary analyses

The rate of positive ethanol tests will be summarized as n (%). Continuous secondary endpoints will be estimated as mean (95% CI), unless they are clearly non-normally distributed, in which case median (range) will be reported. Comparison of endpoints by prostate size, age and duration of symptoms will be examined using regression (linear or logistic, depending on the endpoint).

## Regulatory issues

### Ethical approval

Study was approved by the local ethical committee (EKSG 14/035) and is performed in consideration of the World Medical Association Declaration of Helsinki [23], the guidelines for GCP [24], and the guidelines of the Swiss Academy of Medical Sciences [25]. Handling of all personal data will strictly comply with the federal law of data protection in Switzerland [26].

### Quality control, quality assurance and confidentiality

Trial-related monitoring, audits and regulatory inspections from the ethical committee (EKSG) will be permitted by the principal investigator, providing direct access to source documents. Data collection is performed using electronic case report forms (SecuTrial) programmed by Clinical Trials Unit St. Gallen. Insight into the data collected in this trial will only be provided to the involved investigators and the experts of the ethical committee responsible for the monitoring.

### Missing data

In the case of patients with no follow-up at 12 weeks, baseline and earlier post-operative characteristics will be analyzed, as 12 week control is only a control of functional outcome and does not influence primary study endpoint.

### Safety

The breathalyzer measures ethanol levels on a 10-min interval. New samples will be taken at a 5-min interval if ethanol is detected. The surgeons are blinded to the results of the ethanol measurements but are informed by the anesthetist, if the critical absorption volume of 2 liters is exceeded. This limit will be adopted according to the individual patient’s health condition according to pre- and intraoperative recommendations of the anesthetist. If more than 2 liters of irrigation fluid are absorbed and the patient develops symptoms of cardiopulmonary stress, the operation will be finished and proceeded another day, if infravesical desobstruction was not complete.

## Discussion

The aim of this study is to assess whether absorption of irrigation fluid occurs during TUV-P by expired ethanol breath test. Although different studies have been conducted, using ethanol breath test, the methodology of this monitoring method varies considerably between the studies. Protocol publications such as ours might help to standardize future trial settings.

Using a prospective single-center trial setting with clearly defined endpoints, as well as inclusion and exclusion criteria and study performance according to well-defined quality standards, data will help to assess safety of TUV-P in regard to potentially dangerous absorption of irrigation fluid. Assessment of urinary flow, post void residual and IPPS 12 weeks after the intervention are intended to ensure sufficient operative quality of TUV-P.

Moreover, potential advantages as well as problems of TUV-P can be analyzed. In addition, the study allows comparison to TUR-P data, still representing the gold standard of infravesical desobstruction.

### Trial status

The trial is in the recruiting phase at the time of manuscript submission.

## Abbreviations

BPH: Benign prostate hyperplasia
TUR-P: Transurethral resection of the prostate
TUV-P: Transurethral laser vaporization of the prostate
TUR syndrome: Transurethral resection syndrome
CI: Confidence interval
GCP: Good clinical practice
IPSS: International Prostate Symptom Score
PI: Principle investigator
TRUS: Transrectal ultrasound
